# Changing Trends in the Management of Epistaxis

**DOI:** 10.1155/2015/263987

**Published:** 2015-08-16

**Authors:** Henri Traboulsi, Elie Alam, Usamah Hadi

**Affiliations:** Department of Otolaryngology-Head and Neck Surgery, American University of Beirut Medical Center, Phase I, 6th Floor, Room C-638, Bliss Street, P.O. Box 11-0236, Beirut, Lebanon

## Abstract

Epistaxis is a very common complaint seen by many types of physicians including otolaryngologists, family physicians, and others. Management of epistaxis is often challenging and requires many types of intervention. The following review describes the different types of past and current treatment modalities including cautery, nasal packing, maxillary artery ligation, anterior artery ligation, and sphenopalatine artery ligation. The paper also proposes an algorithm for managing such cases.

## 1. Introduction

Epistaxis is one of the commonest presenting symptoms to ENT physicians as well as to family and emergency physicians. It is thought to affect 10–12% of the population, of which 10% require medical attention [1]. Although most cases are self-limited, some do not resolve without intervention. New treatment options and approaches have developed in the past decade, especially with the advent of nasal endoscopy. The purpose of this paper is to review the different currently available treatment modalities for the management of epistaxis and to propose a comprehensive yet simple and modern algorithm for the treatment of epistaxis. The treatment options will be divided into medical, nonsurgical interventional, and surgical options and will be described along with their advantages, disadvantages, complications, and success/failures rates. The proposed algorithm will argue for an earlier role for surgical intervention with endoscopic ligation of the sphenopalatine artery (ESPAL) in view of recent literature regarding its efficacy, safety, and cost-effectiveness.

## 2. Medical Treatment

Topical decongestants are widely available, and their limited side effect profile makes them a convenient first-line therapy for the treatment of epistaxis. Chart reviews revealed that the use of topical oxymetazoline can be successful in treating posterior epistaxis in the emergency setting in up to 65–75% of cases [2, 3]. They are, however, to be used with caution in hypertensive patients, especially when anxious patients with profuse epistaxis might have significantly elevated blood pressure in the acute setting. Another concern is the drug inability to reach its target areas when the nasal cavity is filled with blood.

Recently, a randomized control trial published by Zahed et al. compared the application of topical tranexamic acid (a drug used for patients with hereditary hemorrhagic telangiectasia) with the use of anterior packing for cases of anterior epistaxis presenting to the emergency department [4]. The study showed that the drug was more efficacious and resulted in more rapid discharge from the emergency department and higher satisfaction rates from patients. A more recent review, however, argued that there is insufficient evidence to date for the use of tranexamic acid in stable patients with spontaneous epistaxis [5].

## 3. Nonsurgical Interventions

### 3.1. Warm Water Irrigation

When nasal packing products came into the market, along with the advent of nasal endoscopy and endoscopic procedures, the technique of warm water irrigation fell out of favor [6]. But later, in 1999, a study by Stangerup et al. showed that warm water irrigation was more effective than nasal packing for the control of posterior epistaxis (55% success rate compared to 44%, resp.) [7]. A more recent article by Novoa and Schlegel-Wagner reports a success rate of 82% in cases of intractable posterior epistaxis with no complications [6]. The group utilize this technique as a first-line treatment for cases of posterior epistaxis. They describe the insertion of a modified bladder catheter that seals the choanae through which water at 50°C will be irrigated with the help of a caloric stimulator and will exit the catheter through a hole proximal to the inflated balloon. It is believed that the warm water causes edema of the nasal mucosa thereby compressing the bleeding vessels in addition to possibly stimulating the coagulation cascade [8].

### 3.2. Cautery via Anterior Rhinoscopy

Initial evaluation of a patient with epistaxis with anterior rhinoscopy might often reveal the source of the bleed if indeed this bleed is anterior.

Cautery options include chemical (with silver nitrate) and electric bipolar cautery. Since chemical cautery is less costly, easier to perform, and more readily available, it is more commonly used, especially by the non-ENT physician. The main risk of this procedure is septal perforation, which increases with bilateral cautery on opposing sides [9].

A recent chart review performed by Shargorodsky et al. reported that 77.1% of anterior epistaxis cases in their case review were treated with silver nitrate cautery with a 79% success rate on the first trial [10].

### 3.3. Nasal Packing

Nasal packing is often an effective and simple means of stopping nasal bleeds. The wide availability of packs, ease of use by nonspecialists, and low cost make this option a valid one as a first-line treatment.

However, nasal packing can be quite uncomfortable and may be responsible for a plethora of complications and adverse effects. Some of these can fortunately be mild and self-limited such as eustachian tube dysfunction, epiphora, and vasovagal reactions during insertion of the pack [11–17]. More importantly, nasal packing can also induce local infections of the nasal cavity and vestibule or can result in more extensive regional infections such as sinusitis and orbital cellulitis [18–21]. A nasal cavity giant pyogenic granuloma has also been described following nasal packs insertion [22]. Rarely, these infections can result in more severe and potentially lethal systemic responses such as toxic shock syndrome and infectious endocarditis [18]. The pressure effect caused by the presence of nasal packs can result in significant complications such as septal abscesses and perforations as well as necrosis of the inferior turbinates [23] and the nasal alae. Fracture of the lamina papyracea and perforation of the palatehave also been described. The presence of the nasal packs has been also shown to disturb normal cardiopulmonary functions and can cause bradycardia and hypoxia [18]. Less commonly, nasal packs can be dislodged from the nasal cavity into the oropharynx resulting in a life-threatening upper airway obstruction [11, 12]. A case of cerebrospinal fluid leak was also reported following the application of a RapidRhino inflatable balloon pack [24]. These severe complications are fortunately rare, but the overall complication rate of nasal packing has been reported to be up to 69% [25].

The failure rate of nasal packing has been reported to be up to 52% [26], and the rate of rebleeding is increased to 70% in patients with bleeding disorders [27]. The traumatic insertion of nasal packs can also cause bleeding in areas different from the one responsible for the primary bleed [28]. These complications and high failure rates make the insertion of nasal packs an extremely unpleasant and often dangerous option for the control of epistaxis.

### 3.4. Embolizations

In an attempt to avoid complications during surgery, angiographic embolization for treating posterior epistaxis has first been described in 1974 [29]. The success of this procedure has been classically reported to be 71–95% [30]. In a recent study comprising 70 patients who underwent angiographic embolization of the sphenopalatine artery, 13% had recurring bleeds within 6 weeks of the procedure and another 14% at a later presentation [31].

The complications of this procedure have been extensively reported in the literature and include hemiplegia, ophthalmoplegia, facial paralysis/paresthesia, blindness, or other neurological deficits caused by accidental embolization of cerebral arteries [18, 32, 33]. These possible complications were shown to occur in up to 27% of cases [18, 20].

Interestingly, some authors advocate embolization of the internal maxillary artery instead of the sphenopalatine artery in children under the age of 10 [34]. Due to these relatively high failure rates and the introduction of the less risky and more successful endoscopic procedures, some advocate the use of angiographic embolization only when endoscopic procedures have failed or are contraindicated [21, 35, 36].

## 4. Surgical Intervention

### 4.1. Maxillary Artery Ligation

In 1965, Chandler and Serrins described the transantral ligation of the maxillary artery under local anesthesia [37]. This technique is classically performed through the Caldwell-Luc approach.

It has been associated with persistent pain in the upper teeth, infraorbital neuralgia, oroantral fistula, sinusitis, potential damage to sphenopalatine ganglion and vidian nerve, and, rarely, blindness [38]. Complications of this approach have been estimated to reach 28% [39].

Chandler and Serrins reported no failures in all 21 patients [37]. A more recent review of the failures of this technique was published in 1988 and reported 15 failures out of 100 patients who underwent the procedure [40]. The authors attributed these failures to the inability to properly locate the IMA and the inability to clip the branches of the internal maxillary artery to the pterygopalatine fossa.

Due to this somewhat invasive approach and potential complications, the transantral ligation of the maxillary artery technique has lost popularity, especially with the advent of the endoscopic procedures.

Ligation of the external carotid artery has also been described for refractory epistaxis; however, its failure was found to be quite high (45%) in a retrospective study conducted in 1992 [41].

### 4.2. Anterior Ethmoid Artery Ligation

The ligation of the anterior ethmoid artery has first been described through a Lynch incision in 1946 [42]. The advances in endoscopic procedures facilitated the development of the endoscopic ligation of this technique. In a recent study [43], a cadaveric dissection examined the feasibility of the procedure as well as the surgical anatomy of the anterior ethmoid artery, which was correctly identified in 98.5% of cases.

A study conducted in 2006 suggested the use of endoscopic anterior ethmoid artery ligation only when the artery is in a mesentery and is clearly visible (present in 20% of cases according to the study). Otherwise, the authors rather suggested an external approach [44].

The surgeon should be familiar with the anatomy of the anterior ethmoid artery and should recognize its intraorbital and ethmoid components in order to properly identify it intraoperatively and to avoid complications, such as bleeding and CSF leak [45, 46]. Interestingly, the anterior ethmoid artery has also been considered to be one of the landmarks for the cranial base [47]. Other reported complications include scarring, edema, facial ecchymosis, and damage to the medial canthal ligament [48].

### 4.3. Endoscopic Nasal Cautery

Cautery under endoscopic vision is another option for the control of epistaxis that may avoid the uncomfortable insertion of nasal packs in the case of an unidentified bleeder. While this can be performed in the operating room, a well-equipped clinic or emergency department can also be adequate settings for this procedure. While some authors report a very high success rate [49], others report a relatively significant risk of failure (17–33%) [21], which may be due to the fact that cauterizing the nasal mucosa may also damage an area that will bleed persistently.

Additionally, nasal cautery for epistaxis has been associated with palatal numbness [50] as well as thermal damage to neural structures, obstruction of the nasolacrimal duct, and trauma of the optic nerve, especially if the patient has previously undergone ethmoidectomy [18].

Cautery of the bleeding nasal mucosa seems to be simple and effective means of epistaxis control; however, the restricted availability of endoscopes and endoscopic surgeons in small centers limits the use of this technique.

### 4.4. Endoscopic Ligation of the Sphenopalatine Artery

The ESPAL was first described over 20 years ago [36]. Interrupting the blood flow in a sufficiently distal area provides an advantage over the previously described techniques by avoiding the possible revascularization from the internal maxillary artery [32].

**Figure 1:**
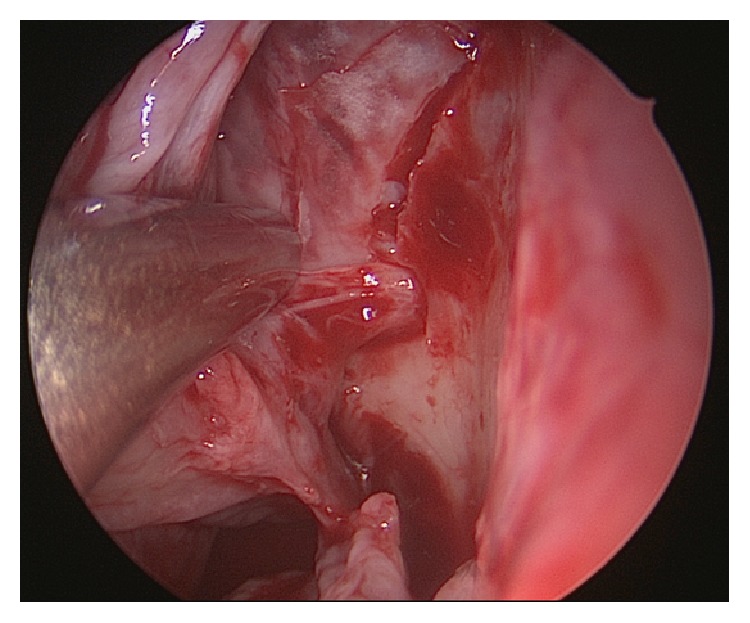
Endoscopic exposure of the left sphenopalatine artery.

Despite being a relatively simple procedure, the endoscopic surgeon should have a good knowledge of the technique and the anatomy of the sphenopalatine artery (SPA) as well as the possible anatomical variations in order to achieve a successful surgery. The SPA is an end branch of the internal maxillary artery and enters the nasal cavity through the sphenopalatine foramen at the posterior lateral nasal wall (Figure 1). It is anteriorly bounded by the crista ethmoidalis, an apparently flawless bony anatomical landmark during surgery [51, 52], which is often taken down to better expose the artery. When the latter or its branches are properly identified, they can be either cauterized or clipped. A study of 67 patients by Nouraei et al. concluded that diathermy is more efficacious than ligation and that not using diathermy was an independent risk factor for failure of the procedure [53].

The branching patterns of the SPA have been extensively studied. It may form two, three, or even four branches [54–56]. However, it appears that two branches are almost consistently present: the posterior lateral nasal artery and the nasal septal branch [54, 55]. Moreover, it seems that the location of the sphenopalatine foramen itself is also variable, for which a classification has been proposed by Wareing and Padgham [56].

**Figure 2:**
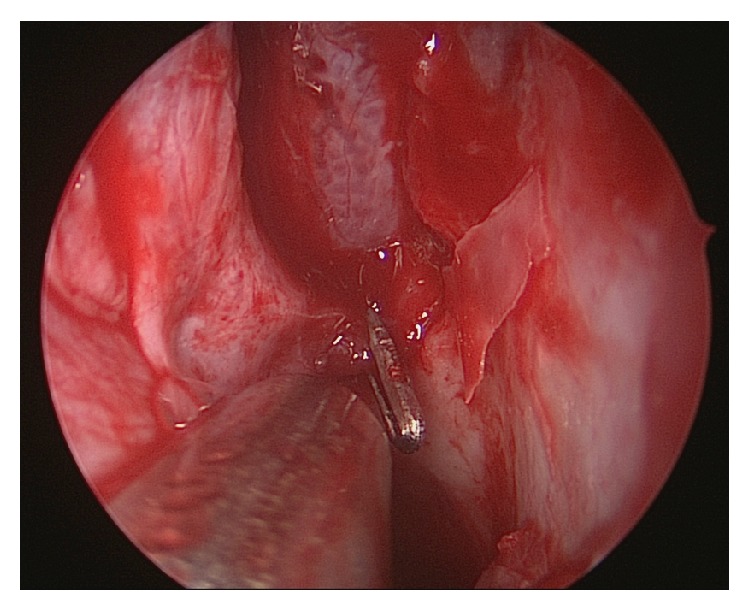
Endoscopic clipping of the left sphenopalatine artery.

If performed correctly in the hands of an experienced endoscopic surgeon, the success rate of this procedure approaches 95–100% [18, 21, 51, 57]. Other authors report a failure rate of 5–10% [39, 58] and early failures are attributed by some to the release of clips or to the failure of identification and clipping of all branches [39] (Figure 2).

The study by Nouraei et al., however, revealed a 90% efficacy rate at 5 years for SPA diathermy. It has also shown that the complication rate has not been associated with any predictive data, such as bilateral surgery, surgery for nasal polyps, or concomitant septoplasty.

A systematic review by Kumar et al. showed that ligation of the SPA and cautery were efficacious in 98% and 100%, respectively [57].

## 5. Discussion of the Proposed Algorithm

The traditional approach to manage patients with intractable epistaxis is to rely on surgery as a last-line treatment once all conservative and nonsurgical treatments (such as nasal packing) have failed. The ease of use of ESPAL technique, its high success rate, and low complication rates have led some authors to propose revision of this management strategy and an earlier deployment of ESPAL. During the past decade, there has been interest in the literature in comparing the cost-effectiveness of ESPAL with other treatment strategies.

A prospective randomized trial by Moshaver et al. in 2004 compared treatment costs of ESPAL with conventional packing. Their reported calculated costs were $5,133 and $12,213, respectively [59].

Additionally, Dedhia et al. conducted a review study in 2013 to determine event probabilities while comparing current practice algorithms (initial nasal pack insertion for 3 days) and first-line ESPAL [60]. Taking into account costs of the respective procedures and the management of recurrences, the authors concluded that the traditional practice arm and first-line ESPAL cost approximately $6,450 and $8,246, respectively. Therefore, according to this study, ESPAL as a first-line treatment for epistaxis might actually be more cost-effective than traditional approaches that rely on prolonged insertion of nasal packs initially.

Similarly, a study conducted by Rudmik and Leung in 2014 compared the cost-effectiveness of ESPAL and embolization for intractable epistaxis, defined as failure of posterior nasal packing after 3 days [61]. Taking incremental cost-effectiveness ratio (ICER) as an outcome measure and a modeling-based economic evaluation using a decision tree analysis to incorporate postprocedural outcomes, the authors concluded that embolization was more costly compared to ESPAL ($22,324.70 and $12,484.14, resp.). The time horizon of the decision tree analysis was 2 weeks, and a multivariate sensitivity analysis confirmed that this economic conclusion was correct at a 74% certainty at least.

More recently, the same group published a modeling-based simulation of a 50-year-old male with intractable epistaxis [62]. The risk model took into account the probabilities of the complications of each intervention, in 6 laddered management algorithms, using posterior packing, embolization, and ESPAL, in different sequences. The severity of each complication was monetized. They found that all 6 laddered strategies would achieve 99% success rate after 2 interventions; however, ESPAL and embolization were more likely to succeed after a single procedure. Strategies starting with packing and ESPAL had the lower risk.

When combining the results of this risk analysis with data on cost-effectiveness, the authors advocated a laddered approach to intractable epistaxis starting with ESPAL first.

In addition to that, there are other advantages of ESPAL over embolization, which include a reduced risk of major complications (such as stroke and blindness), direct endoscopic visualization of the bleeding site, potential diagnosis of rare causes of bleeding such as neoplasms with the possibility of biopsy, an opportunity to perform a concurrent anterior ethmoid artery ligation if required, and a reported lower health care cost [63].

On the other hand, many patients only experience one episode of epistaxis which may never recur, while others have only mild anterior epistaxis that may only require minimal definitive intervention. It would be difficult to justify the costs and the risks of surgery for these patients.

**Figure 3:**
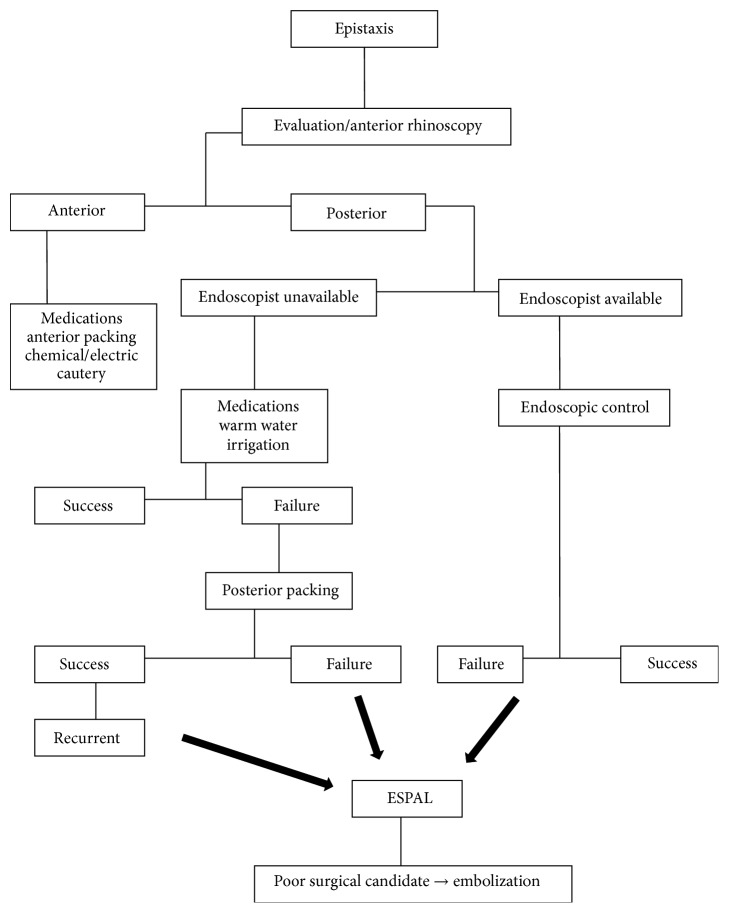
Algorithm for the management of epistaxis.

Therefore, we suggest in our algorithm (Figure 3) treating mild cases of anterior epistaxis with traditional and conservative measures (mentioned above).

The management of posterior nasal bleeds will depend on the availability of experienced endoscopists and relevant equipment. The experienced endoscopist may be successful in treating these patients in an emergency setting, therefore avoiding the potential adverse effects of packs insertion and the potential complications and costs of surgery under general anesthesia. ESPAL can always be done after failure of this procedure.

When an endoscopist is not available, medical therapy or warm water irrigation can be attempted before posterior nasal packing. Recurrent cases, or failures of nasal packing, should be referred to an endoscopist for ESPAL. Endovascular embolization can be performed under local anesthesia and can be considered an alternative to ESPAL if patients are poor surgical candidates.

## 6. Conclusion

The management of epistaxis enjoys a wide range of strategies and treatment options. However, it is important to appreciate when to correctly employ the different individual interventions. It is also important to involve an experienced endoscopist when appropriate who can intervene either with endoscopic control in the emergency department or with ESPAL in the operating room. Recent literature advocates an earlier surgical intervention with ESPAL for such cases due to its simplicity, high success rate, low risks, and cost-effectiveness compared to other treatment modalities such as posterior nasal packing.
